# Short-term serial assessment of electronic patient-reported outcome for depression and anxiety in breast Cancer

**DOI:** 10.1186/s12885-021-08771-y

**Published:** 2021-09-29

**Authors:** Jeeyeon Lee, Jin Hyang Jung, Wan Wook Kim, Byeongju Kang, Jungmin Woo, Hyo-Deog Rim, Yee Soo Chae, Soo Jung Lee, Gi Hwan Kim, Won Kee Lee, Ho Yong Park

**Affiliations:** 1 Department of Surgery, School of Medicine, Kyungpook National University, Kyungpook National University Chilgok Hospital, Daegu, 41404 Republic of Korea; 2 Department of Psychiatry, School of Medicine, Kyungpook National University, Kyungpook National University Hospital, Daegu, Republic of Korea; 3 Department of Oncology/Hematology, School of Medicine, Kyungpook National University, Kyungpook National University Chilgok Hospital, Daegu, Republic of Korea; 4 Clupea, Inc., Daegu, Republic of Korea; 5 Department of Medical Informatics, School of Medicine, KyungPook National University, Daegu, Republic of Korea

**Keywords:** Breast cancer, Depression, Anxiety, Serial assessment

## Abstract

**Purpose:**

The incidence of depression and anxiety is higher in patients with breast cancer than in the general population. We evaluated the degree of depression and anxiety and investigated the changes in patients with breast cancer during the treatment period and short-term follow-up period.

**Methods:**

Overall, 137 patients with breast cancer were evaluated using the Patient Health Questionnaire 9-item depression scale (PHQ-9) and Generalized Anxiety Disorder scale (GAD-7). The scales were developed as a web-based electronic patient-reported outcome measure, and serial results were assessed before the operation, after the operation, in the post-treatment period, and in the 6-month follow-up period after surgery.

**Results:**

The degree of depression and anxiety increased during treatment and decreased at 6-month follow-up, even if there were no statistical differences among the four periods (PHQ-9: *p* = 0.128; GAD-7: *p* = 0.786). However, daily fatigue (PHQ-9 Q4) and insomnia (PHQ-9 Q3) were the most serious problems encountered during treatment and at 6-month follow-up, respectively. In the GAD-7, worrying too much (Q3) consistently showed the highest scores during the treatment and follow-up periods. Of the patients, 7 (5.11%) and 11 (8.03%) patients had a worsened state of depression and anxiety, respectively, after treatment compared with before treatment.

**Conclusion:**

Most factors associated with depression and anxiety improved after treatment. However, factors such as insomnia and worrying too much still disturbed patients with breast cancer, even at 6-month follow-up. Therefore, serial assessment of depression and anxiety is necessary for such patients.

**Supplementary Information:**

The online version contains supplementary material available at 10.1186/s12885-021-08771-y.

## Introduction

Breast cancer is the most common malignancy in middle-aged Korean women; it is more frequently associated with depression and anxiety than other malignancies [1–4]. Physicians and patients believe that their mental stress would spontaneously improve after the treatment is finished; however, mental status should be managed actively because poor mental health may negatively affect patient compliance and tumor prognosis [5, 6].

Recently, the psycho-oncological therapeutic approach has become an essential tool in the care of cancer survivors, and there has been much interest in the quality of life and mental health of cancer survivors [7]. Although a previous study reported that depression and anxiety improve with time after the diagnosis of breast cancer, no serial study related to the treatment periods of breast cancer has been conducted [8]. Therefore, it is difficult to predict at what point physicians should intervene to improve the patient’s mental health.

The patient-reported outcome (PRO) plays a crucial role in the assessment of the patient’s own health and is the best way to obtain an accurate assessment [9]. Because it is important that a serial assessment be easily accessible, the use of an electronic PRO (ePRO) platform, such as a web-based or smartphone-based application, is preferred in younger generation. Furthermore, the use of an ePRO can minimize the time spent for statistical analysis and maximize the number of responders with early intervention.

The authors evaluated the degree of and observed changes in anxiety and depressive symptoms throughout the preoperative, postoperative, post-treatment, and 6-month follow-up periods using the ePRO-based Patient Health Questionnaire 9-item depression scale (PHQ-9) and Generalized Anxiety Disorder scale (GAD-7) to figure out the candidates who may need a psychotherapeutic management [10, 11].

## Methods

Authors from the breast cancer center of Kyungpook National University Chilgok Hospital (KNUCH, Daegu, Republic of Korea) designed the SAAD trial (Serial Assessment of Anxiety and Depressive symptoms in breast cancer), which is a prospective, questionnaire-based serial cohort study. The ePRO platform was developed, and archived data was analyzed by Clupea, Inc. (epro.clupea.kr) to evaluate depressive and anxiety symptoms of patients with breast cancer.

Because depressive mood and anxiety may become worse with the operation date approaching, the first examination was performed on the first day of visit to our center, and the postoperative examination was performed during the hospitalization period within 1 week after operation. However, the survey was not conducted immediately after surgery because postoperative pain would affect the results. Moreover, post-treatment examinations were conducted at the visit to the breast clinic after completion of chemotherapy or radiation and at 6-month follow-up examination after the surgery on the day of checking results of regular surveillance.

The study was conducted from January to December 2020, and all procedures in this study involving human participants were performed in accordance with the ethical standards of the Institutional Review Board of the KNUCH (KNUCH 2020–06–029-001). All enrolled patients signed the informed consent.

### Patients

In total, 182 patients were diagnosed with operable breast cancer between January and April 2020. Among them, 12 patients refused to participate in the study, and 33 patients did not complete the survey for four times during the treatment and follow-up period. Eventually, 137 patients with breast cancer who had received surgery and additional treatment at KNUCH were included in this study. Patients were administered an electronic survey four times: preoperative, postoperative, post-treatment (after finishing adjuvant treatment, except for hormone treatment), and at 6-month follow-up from surgery. When patients were diagnosed with depression or anxiety disorder by a psychiatrist before the diagnosis of breast cancer, they were excluded from the study owing to potential different effects.

When the physician created a patients’ list on the web-based system, a secured login was activated using the patients’ ID numbers and names. Patients logged in using personal computer, tablet, or their own smartphone. The patients clicked the answers by themselves, and the total scores of the PHQ-9 and GAD-7 were shown after completion of the questionnaires. The physicians checked the mental status of patients in real time.

Medical records were reviewed to determine clinical and pathological factors, including age, body mass index, menstruation, marital status, childbearing status, family history of breast or ovarian cancer, and history of hormone replacement therapy as well as clinicopathological characteristics, including clinical and pathologic tumor size, axillary lymph node status, tumor subtype, adjuvant treatment, and type of surgery.

### Depression and anxiety disorder scale

The ePRO was constructed based on the PHQ-9 and GAD-7. We compared and analyzed the changes in total scores and the scores for each question on the PHQ-9 and GAD-7.

The self-administrated PHQ is a standard scale that assesses several psychiatric disorders, including depressive disorder, panic disorder, anxiety disorder, bulimia nervosa, and other minor disorders (5–8). However, the brief form of the PHQ with the nine-item depression module, the so-called PHQ-9, is generally used to screen for depression [12–16]. The total score of the PHQ-9 ranges from 0 to 27, and the level of depression severity can be classified as 0–4 (no or minimal depressive symptoms), 5–9 (mild depressive symptoms), 10–19 (moderate depressive symptoms), and 20–27 (severe depressive symptoms).

Anxiety symptoms were measured using the GAD-7, a self-administrated questionnaire, which comprises seven brief items [17–19]. Each item is scored 0 (*not at all*) to 3 (*nearly every day*), and the threshold points of mild, moderate, and severe anxiety are 5, 10, and 15, respectively.

### Development of the ePRO

The ePRO program was created to measure the depression and anxiety scale before and after treatment for patients with breast cancer (Supplementary figure 1). The PHQ-9 and GAD-7 were used as tools to evaluate symptoms of depression and anxiety, and the questionnaire was developed as a web-based platform considering the user’s convenience (Supplementary figure 2).

After logging in to the main web page for the first time, the examiner activates the patient’s name with brief clinical information to reduce the inconvenience. When the questionnaire is completed, the inspectors can check the results of each question, and serial results are archived. The severity of each item in the PHQ-9 and GAD-7 is expressed in the degree of green and pink colors, respectively (Fig. 2). In addition, to enable the examiner to detect abnormalities at a glance, severity is expressed as follows: no bar = no depression (total PHQ-9 score 0–4); light green bar = mild depression (total PHQ-9 score 5–9); green bar = moderate depression (total PHQ-9 score 10–19); dark green bar = severe depression (total PHQ-9 score 20–27); no bar = no anxiety (total GAD-7 score 0–4); light pink bar = mild anxiety (total GAD-7 score 5–9); pink bar = moderate anxiety (total GAD-7 score 10–14); and hot pink bar = severe anxiety (total GAD-7 score 15–21).

The correlation graphs between depression and anxiety were obtained as a quartile, a one-dimensional correlation, and a heat map–style graph (Supplementary figure 3). Changes in the degree of depression and anxiety were defined as either “improved” or “worsened.”

### Statistical analysis

The mean scores of PHQ-9 and GAD-7 scales were compared based on the results of test which were conducted before surgery (preoperative), after surgery (postoperative), after adjuvant treatment (post-treatment) except hormone treatment, and at the 6-month follow-up. In addition, each questionnaire items were analyzed and compared, and the changes in the degree of depression and anxiety were analyzed.

The significance of the mean difference among the four groups was tested through repeated measures of ANOVA, and if it was significant, post hoc comparison between the two groups was performed using the Bonferroni correction after obtaining the significance probability with the least-squares mean method.

## Results

The mean age of the 137 patients was 53.83 years (SD, ±11.6 years), and the mean body mass index was 24.82 kg/m^2^ (SD, ±7.25). The mean age at menarche was 15.09 years (SD, ±3.25), and 65 patients (47.45%) were in the postmenopausal state. Overall, 106 patients (77.37%) were married, 25 patients (18.25%) were single, and 6 patients (4.38%) were divorced. Sixteen patients (11.68%) did not have a child, and 30 patients (21.90%) had > 3 children.

Patients underwent breast-conserving surgery (*n* = 98; 71.54%) or mastectomy (*n* = 39; 28.47%), and 42 patients (30.66%) received immediate breast reconstruction. More than 70% of patients had early breast cancer (less than stage I), and > 80% of patients had hormone-positive breast cancer. Sixty-two patients (45.26%) and 97 patients (70.81%) received adjuvant chemotherapy and radiotherapy, respectively (Table 1).

**Table 1:** Clinicopathological characteristics of patients with breast cancer

	*n* = 137
Age (mean ± SD, years)	53.83 ± 11.60
Body mass index (mean ± SD, kg/m^2^)	24.82 ± 7.25
Age of menarche (mean ± SD, years)	18.81 ± 0.71
Postmenopausal status	65 (47.45)
Marital status	
Married	106 (77.37)
Single	25 (18.25)
Divorced	6 (4.38)
Number of children	
0	16 (11.68)
1	18 (13.14)
2	73 (53.28)
> 3	30 (21.90)
Family history of breast or ovarian cancer	6 (4.38)
History of hormone replacement therapy	
No	134 (97.81)
Yes	3 (2.19)
Location of tumor	
Right	76 (55.48)
Left	61 (44.53)
Types of tumor	
Ductal carcinoma in situ	21 (15.33)
Invasive ductal carcinoma	112 (81.76)
Invasive lobular carcinoma	1 (0.73)
Other	3 (2.19)
Pathologic stage	
0	22 (16.06)
IA	66 (48.18)
IIA	25 (18.25)
IIB	15 (10.95)
IIIA	7 (5.11)
IIIC	2 (1.46)
Estrogen receptor	
Positive	119 (86.87)
Negative	18 (13.14)
Progesterone receptor	
Positive	102 (74.46)
Negative	35 (25.55)
Her2/neu gene	
Positive	24 (17.52)
Negative	113 (82.49)
Triple negative breast cancer	17 (12.41)
Ki67 index	
≥ 15%	100 (73.00)
< 15%	37 (27.01)
Type of breast surgery	
Breast conserving surgery	98 (71.54)
Mastectomy	39 (28.47)
Breast reconstruction	42 (30.66)
Type of axillary surgery	
No operation	4 (2.92)
Sentinel lymph nodes biopsy	119 (86.87)
Axillary lymph nodes dissection	14 (10.22)
Adjuvant chemotherapy	62 (45.26)
Adjuvant radiotherapy	97 (70.81)
Adjuvant hormonal therapy	93 (67.89)
Follow-up period (mean ± SD, months)	8.01 ± 0.69

^*^Data are expressed as n, %

**Fig. 1:**
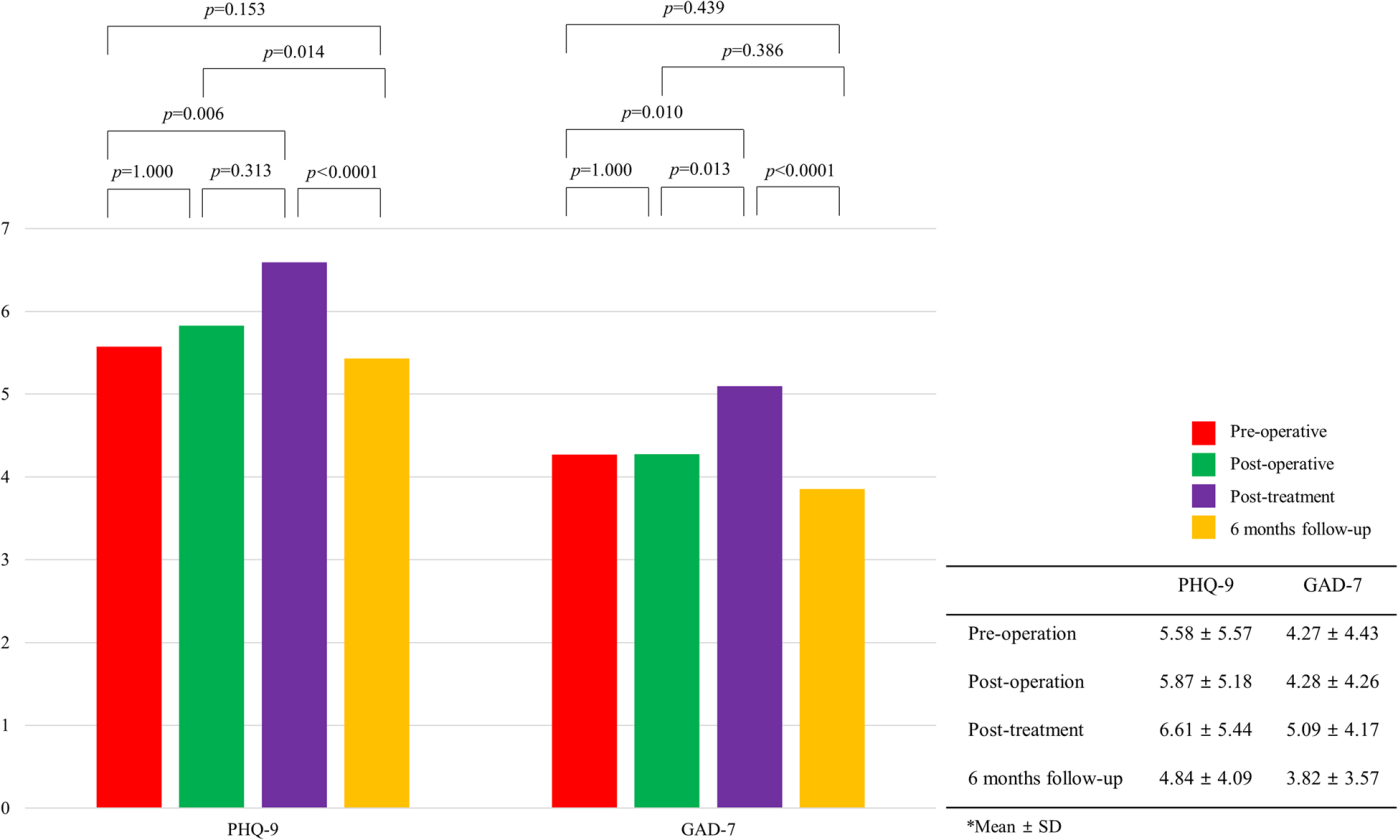
Mean scores of the PHQ-9 and GAD-7 in the preoperative, postoperative, post-treatment, and 6-month follow-up periods in patients with breast cancer

The mean scores of both the PHQ-9 and GAD-7 increased during treatments, including surgery and adjuvant treatments, and the scores decreased at the time of the 6-month follow-up period compared with during the treatment period (Fig. 1). The mean score of PHQ-9 significantly decreased at the 6-month follow-up period compared with those in the postoperative and post-treatment periods (*p* < 0.0001), and the mean score of GAD-7 significantly decreased at the 6-month follow-up period compared with that in the post-treatment period (*p* < 0.0001).

**Fig. 2:**
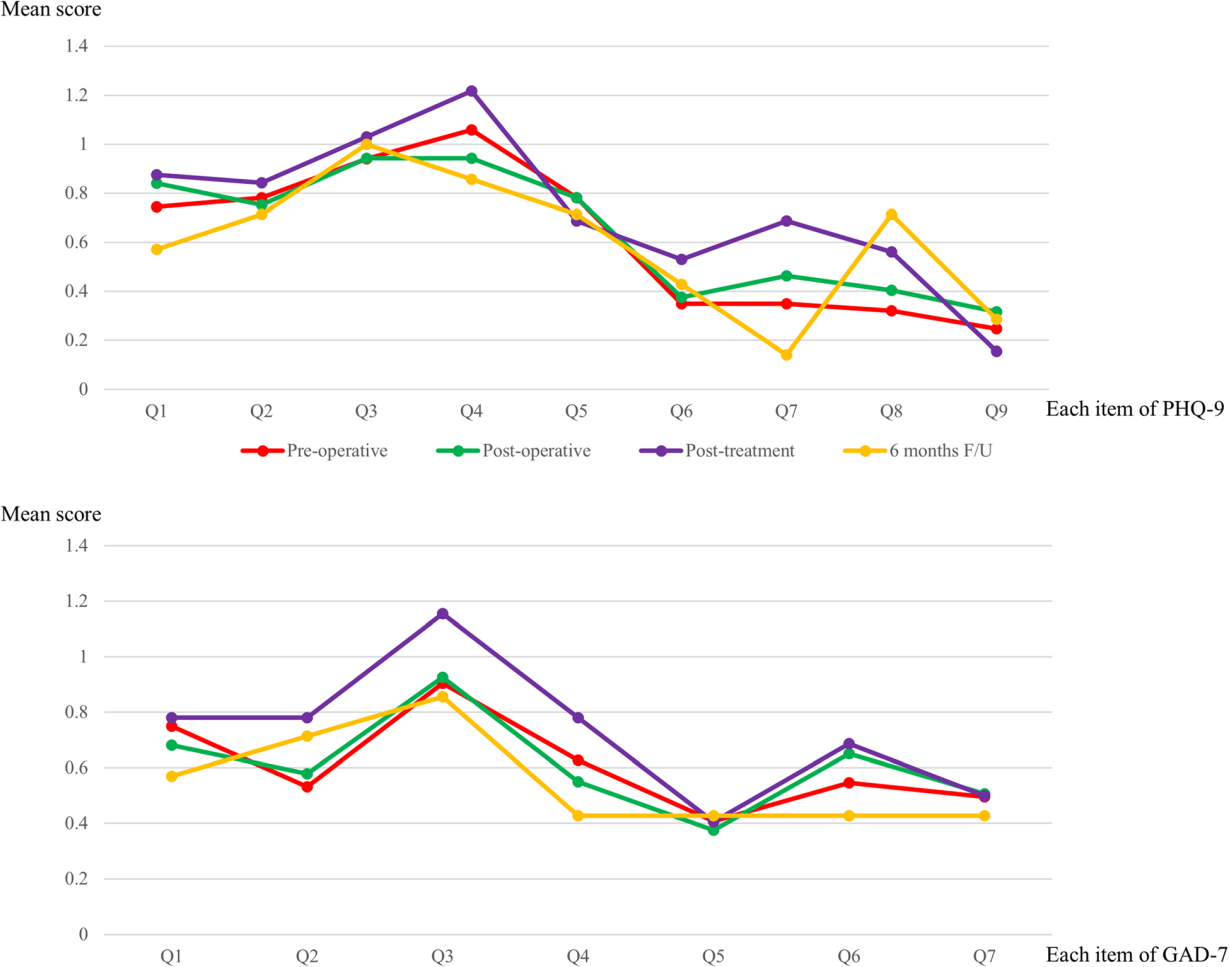
Mean scores of each item on the PHQ-9 (**a**) and GAD-7 (**b**)

However, the mean scores of each item were different according to the content of the questions. For the PHQ-9, daily fatigue (PHQ-9 Q4) was the most serious problem encountered during the treatment period, whereas the largest number of patients complained about insomnia (PHQ-9 Q3) and second largest number of patients complained about being fidgety or restless (PHQ-9 Q8) at 6 months after the operation. For the GAD-7, worrying too much (GAD-7 Q3) consistently showed the highest scores during the treatment and follow-up periods (Fig. 2). However, there were no statistical differences among the four periods for each item of PHQ-9 and GAD-7 (Table 2).

**Table 2:** Statistical differences among four periods in each item of PHQ-9 and GAD-7

PHQ-9	Preoperative	Postoperative	Post-treatment	6 months after surgery	*p*-value
Q1. Little interest or pleasure in doing things?	0.75 ± 0.96	0.84 ± 0.78	0.88 ± 0.94	0.57 ± 0.49	0.739
Q2. Feeling down, depressed, or hopeless?	0.79 ± 0.87	0.75 ± 0.77	0.84 ± 0.85	0.71 ± 0.70	0.961
Q3. Trouble falling or staying asleep or sleeping too much?	0.96 ± 1.00	0.94 ± 0.92	1.03 ± 1.09	1.00 ± 1.07	0.971
Q4. Feeling tired or having little energy?	1.08 ± 1.02	0.94 ± 0.86	1.22 ± 0.87	0.86 ± 0.99	0.546
Q5. Poor appetite or overeating?	0.81 ± 0.95	0.78 ± 0.87	0.69 ± 0.82	0.71 ± 0.88	0.957
Q6. Feeling bad about yourself or that you are a failure or have let yourself or your family down?	0.39 ± 0.07	0.38 ± 0.67	0.53 ± 0.76	0.43 ± 1.05	0.638
Q7. Trouble concentrating on things, such as reading the newspaper or watching television?	0.66 ± 0.40	0.46 ± 0.65	0.69 ± 0.93	0.14 ± 0.35	0.060
Q8. Moving or speaking so slowly that other people could have noticed? Or so fidgety or restless that you have been moving a lot more than usual?	0.38 ± 0.74	0.41 ± 0.69	0.56 ± 0.84	0.71 ± 1.03	0.246
Q9. Thoughts that you would be better off dead or thoughts of hurting yourself in some way?	0.31 ± 0.63	0.32 ± 0.65	0.16 ± 0.45	0.29 ± 0.70	0.664
GAD-7					
Q1. Feeling nervous, anxious, or on edge	0.75 ± 0.76	0.68 ± 0.68	0.78 ± 0.79	0.57 ± 1.05	0.833
Q2. unable to stop or control worrying	0.53 ± 0.75	0.58 ± 0.74	0.78 ± 0.83	0.71 ± 1.03	0.405
Q3. Worrying too much about different things	0.91 ± 0.84	0.93 ± 0.81	1.16 ± 0.95	0.86 ± 0.99	0.502
Q4. Trouble relaxing	0.63 ± 0.84	0.55 ± 0.80	0.78 ± 0.87	0.43 ± 0.73	0.559
Q5. Being so restless that it is hard to sit still	0.41 ± 0.76	0.38 ± 0.60	0.41 ± 0.56	0.43 ± 0.73	0.990
Q6. Becoming easily annoyed or irritable	0.55 ± 0.80	0.65 ± 0.84	0.69 ± 0.78	0.43 ± 1.05	0.686
Q7. Feeling afraid as if something awful might happen	0.50 ± 0.75	0.51 ± 0.68	0.50 ± 0.72	0.43 ± 0.73	0.995

**Fig. 3:**
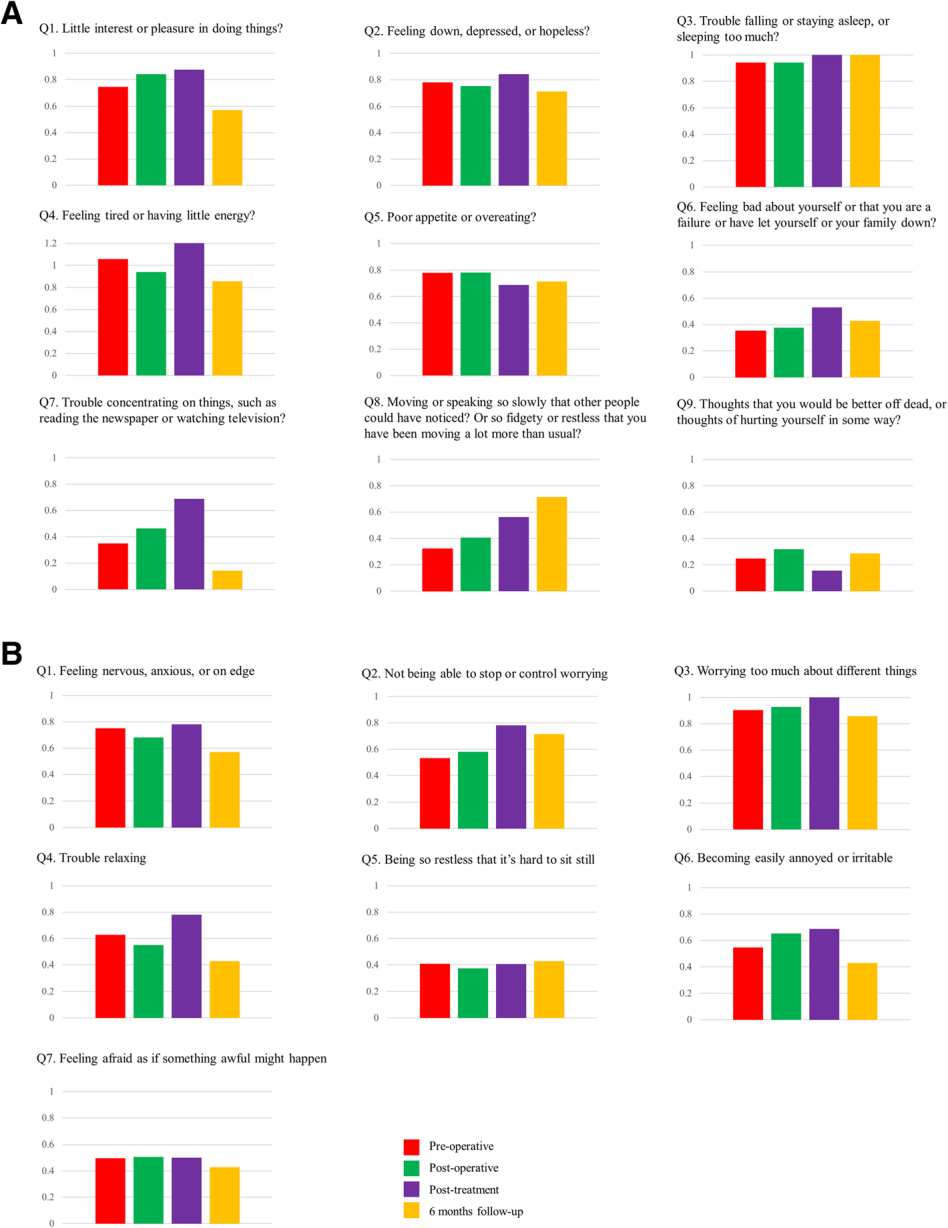
Scores of each item on the PHQ-9 (**a**) and GAD-7 (**b**). Most of the specific symptoms were improved at the 6-month follow-up period. All contents of the system were originally written in Korean

Based on analysis of the trend of each item, most statuses of the PHQ-9 (Q1, Q2, Q4, Q5, Q6, Q7, and Q9) and GAD-7 (Q1, Q3, Q4, Q6, Q7) improved at the 6-month follow-up. However, insomnia (PHQ-9 Q3) and feeling fidgety or restless (PHQ-9 Q8) were still disturbing the patients at 6 months (Fig. 3). Compared with the status of depression and anxiety between the pre-treatment and post-treatment periods, the degrees of depression and anxiety improved in 27 patients (19.71%) and 26 patients (18.98%), respectively. On the contrary, 7 patients (5.11%) and 11 patients (8.03%) showed worsened depression and anxiety status (Table 3). The changes of PHQ-9 and GAD-7 score in each patient are shown in supplementary Figure 4.

**Table 3:** Degree of depression and anxiety in pretreatment and post-treatment states for breast cancer

			Pretreatment state									
			Depression						Anxiety			
			No	Mild	Moderate	Severe			No	Mild	Moderate	Severe
Post-treatment state	Depression	No	66 (48.18)	21 (15.33)	2 (1.46)	–	Anxiety	No	75 (54.75)	20 (14.6)	4 (2.92)	–
		Mild	4 (2.92)	18 (13.14)	3 (2.19)	1 (0.73)		Mild	7 (5.11)	9 (6.57)	1 (0.73)	–
		Moderate	–	3 (2.19)	11 (8.03)	7 (5.11)		Moderate	1 (0.73)	3 (2.19)	5 (3.65)	1 (0.73)
		Severe	–	–	–	1 (0.73)		Severe	2 (1.46)	2 (1.46)	3 (2.19)	4 (2.92)
Improved		34 (24.82)					26 (18.98)					
Worsen		7 (5.11)					18 (13.14)					

^*^Data are expressed as n, %

## Discussion

The treatment process of various malignant tumors occasionally results in anxiety or depression in cancer survivors, from diagnosis to surveillance [20]. The PHQ-9 and GAD-7, which are well-validated, are the most commonly used measures for the objective assessment of depression and anxiety, respectively [11, 14, 17, 20, 21]. In recent study, the authors evaluated changes in depression and anxiety during treatment and the short-term follow-up period in patients with breast cancer. Although the degree of depression and anxiety was maintained or increased during breast cancer treatment, it decreased during the 6-month follow-up period after surgery than during the treatment period. This could be the reason why physicians and caregivers need to continuously support patients with breast cancer regarding their mental problems and stress during treatment. There were significant differences during the four periods, including preoperative, postoperative, post-treatment, and 6-month follow-up periods, and the degree of depression was significantly higher at the immediate post-treatment period than during the postoperative and 6-month follow-up period.

Regarding each question in PHQ-9 and GAD-7, the Q3 (trouble falling or staying asleep, or sleeping too much?) and Q4 (feeling tired or having little energy?) of PHQ-9 and Q3 (worrying too much about different things) of GAD-7 showed highest mean scores for all four periods. At the 6-month follow-up, the treatments were exhausted, except hormone treatment, and most factors had improved, including depression and anxiety. However, the sleep disturbance (Q3) of PHQ-9 was determined to be the most severe and persisting problem until 6 months after the completion of breast cancer treatment and worrying about many things (Q3) of GAD-7 persisted with higher scores in every time period, although Burgess et al. reported that the prevalence of depression, anxiety, or both decreases and the survival period passes [8].

For accurate evaluation of patients’ mental health status, it is more effective when the test questionnaires are answered by the patients themselves because of its reliability, objectiveness, and accuracy. However, the patients’ burden, such as long repeated work or too long sentences, has been indicated as a reason for noncompliance. The elderly or sicker population showed lower compliance than the healthier population [22–26]. Although many studies have measured depression and anxiety in patients with breast cancer at a specific treatment or time point, only few studies have used continuous follow-up tests [8, 27]. The authors conducted a serial assessment, which was performed using the ePRO platform for depressive mood and anxiety in patients with breast cancer during treatment and short-term follow-up periods. We found that the depressive mood and anxiety gradually increased and improved when the treatments are over.

The main limitation of this study was that we were unable to evaluate the degree of depression and anxiety at the time of each treatment, such as chemotherapy and radiotherapy. Although Nakamura et al. [23] reported that chemotherapy has a decisive effect on depression and anxiety in patients with breast cancer, we could not assess this effect at each time points because the chemotherapy and radiotherapy were performed in other departments based on the multidisciplinary clinical system. And we performed the study with only small population and did not consider the effects of each treatment on mental health status. Furthermore, we did not conduct the analysis for within-individual comparison. However, we conducted this study using a web-based system for patients with breast cancer amd assessed depression and anxiety at least four consecutive times in patients with breast cancer. Clinging to the classical method of assessment without considering the characteristics of modern patients degrades the reliability and accuracy of the tests. Furthermore, it would be unreasonable to reach a conclusion regarding a patient’s mental health status using the result of only a single test point. Therefore, for more accurate evaluation of the mental health status of patients with breast cancer, further serial assessment of depression and anxiety is necessary using a more accessible system, and it may lead to an improvement in their quality of life.

## Conclusion

According to the short-term serial assessment of depression and anxiety, most factors associated with depression and anxiety improved after treatment. However, sleep disturbance and worrying about several things persisted with higher scores at every time period, even at the 6-month follow-up. Therefore, patients with breast cancer should be evaluated for depression and anxiety continuously as well as during the treatment period, and patients and physicians should work together to actively manage them.

## Supplementary Information

**Additional file 1: Supple Figure 1.** Flow chart of the electronic patient-reported outcome (ePRO) of the PHQ-9 and GAD-7 in breast cancer.

**Additional file 2: Supple Figure 2.** Patient list from the electronic patient-reported outcome (ePRO) of anxiety and depressive symptoms for patients with breast cancer. The severity of each item in the PHQ-9 and GAD-7 is expressed in degrees of green and pink colors, respectively.

**Additional file 3: Supple Figure 3.** Correlation graphs between depression and anxiety as a quartile, one-dimensional correlation, and a heat map–style graph.

**Additional file 4.**

## Data Availability

The data sets generated and/or analyzed in this study are not publicly available. However, they are available from the corresponding author upon reasonable request.

## Abbreviations

PRO: Patient-reported outcome
ePRO: electronic patient-reported outcome
PHQ-9: Patient Health Questionnaire 9-item depression scale
GAD-7: Generalized Anxiety Disorder scale (GAD-7)
