# Supplementary figures and images for: Short-term serial assessment of electronic patient-reported outcome for depression and anxiety in breast Cancer
Source: BMC Cancer. 2021 Sep 29;21:1065. doi: 10.1186/s12885-021-08771-y (PMC8479978; doi:10.1186/s12885-021-08771-y)

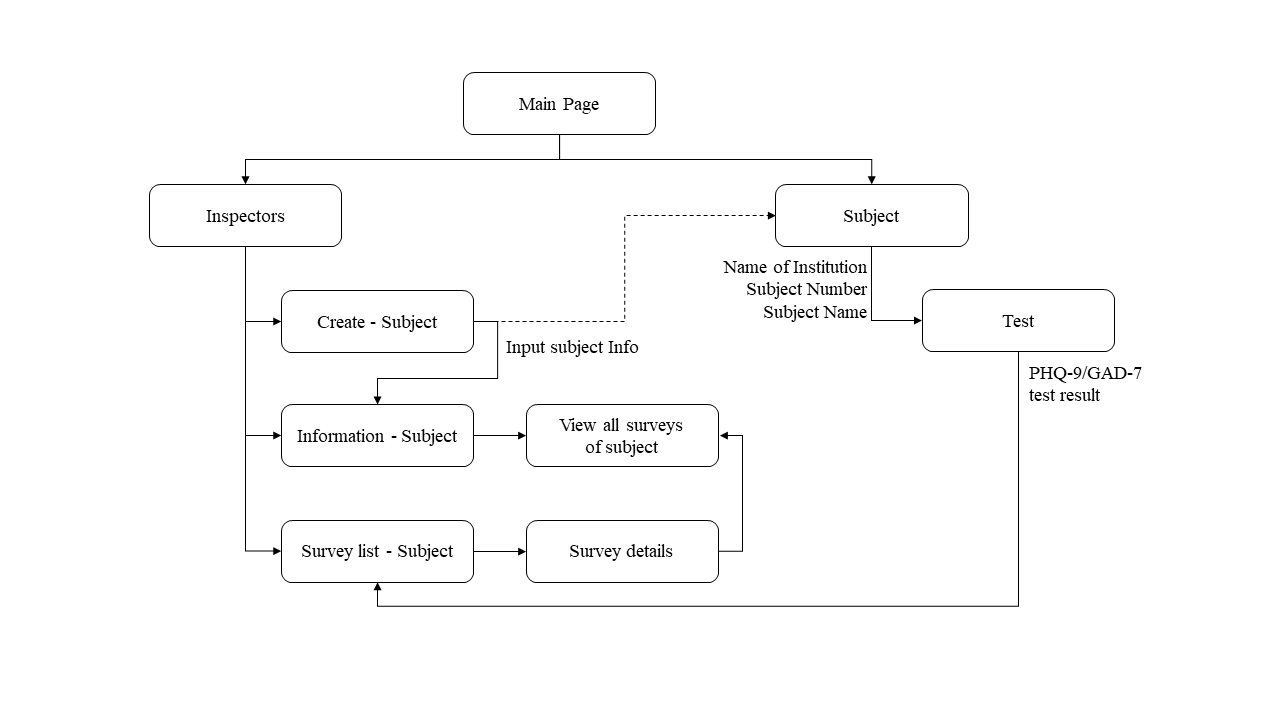

Supplement: Supplementary file 1 — Additional file 1: Supple Figure 1. Flow chart of the electronic patient-reported outcome (ePRO) of the PHQ-9 and GAD-7 in breast cancer. [file 12885_2021_8771_MOESM1_ESM.tif]

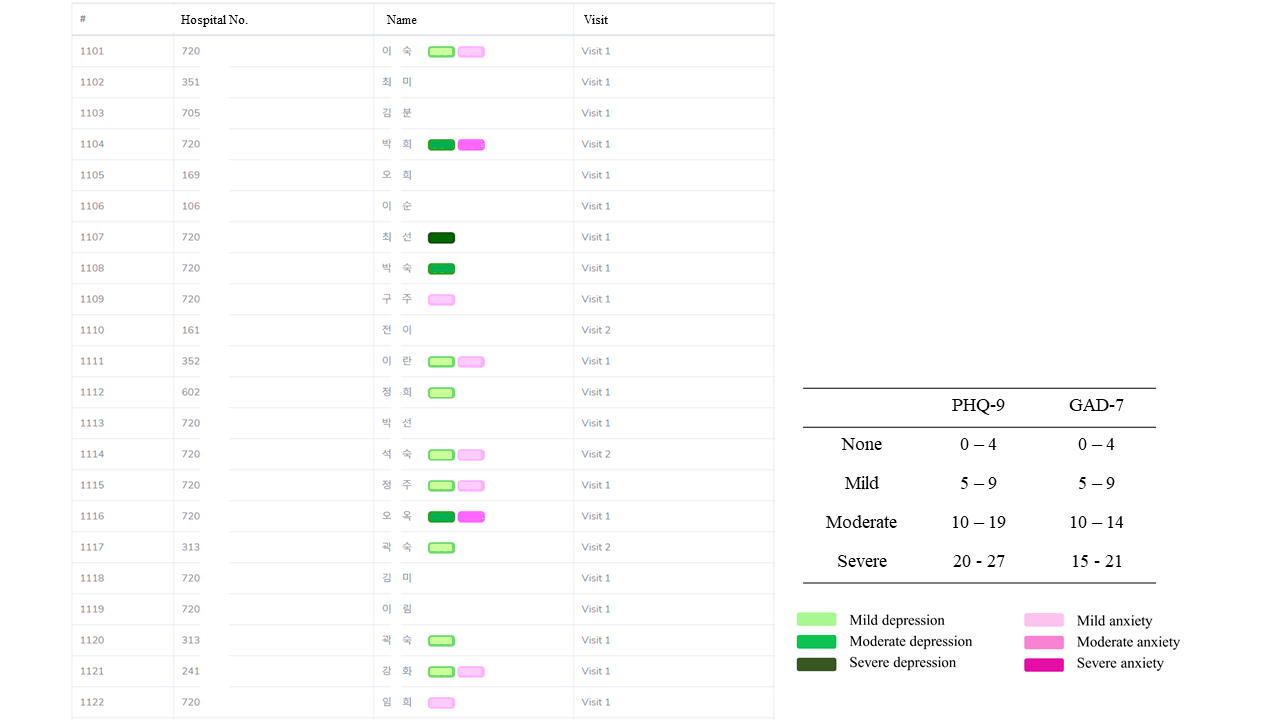

Supplement: Supplementary file 2 — Additional file 2: Supple Figure 2. Patient list from the electronic patient-reported outcome (ePRO) of anxiety and depressive symptoms for patients with breast cancer. The severity of each item in the PHQ-9 and GAD-7 is expressed in degrees of green and pink colors, respectively. [file 12885_2021_8771_MOESM2_ESM.tif]

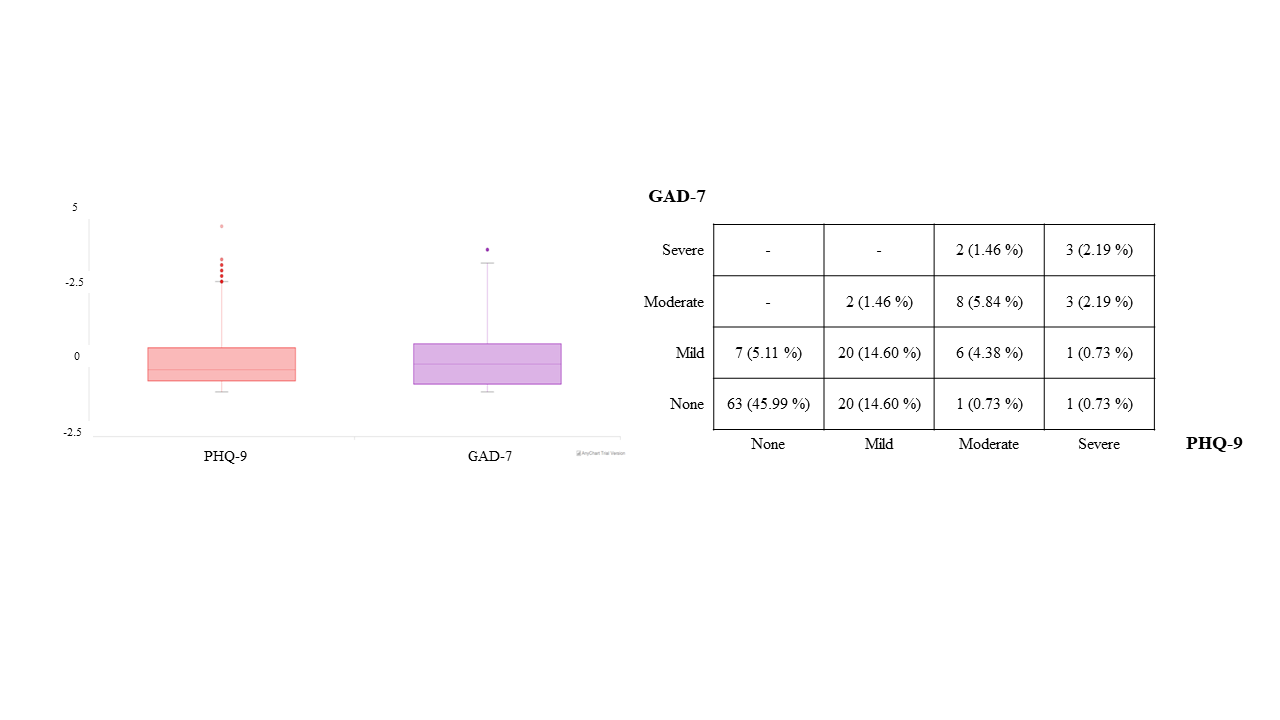

Supplement: Supplementary file 3 — Additional file 3: Supple Figure 3. Correlation graphs between depression and anxiety as a quartile, one-dimensional correlation, and a heat map–style graph. [file 12885_2021_8771_MOESM3_ESM.tif]

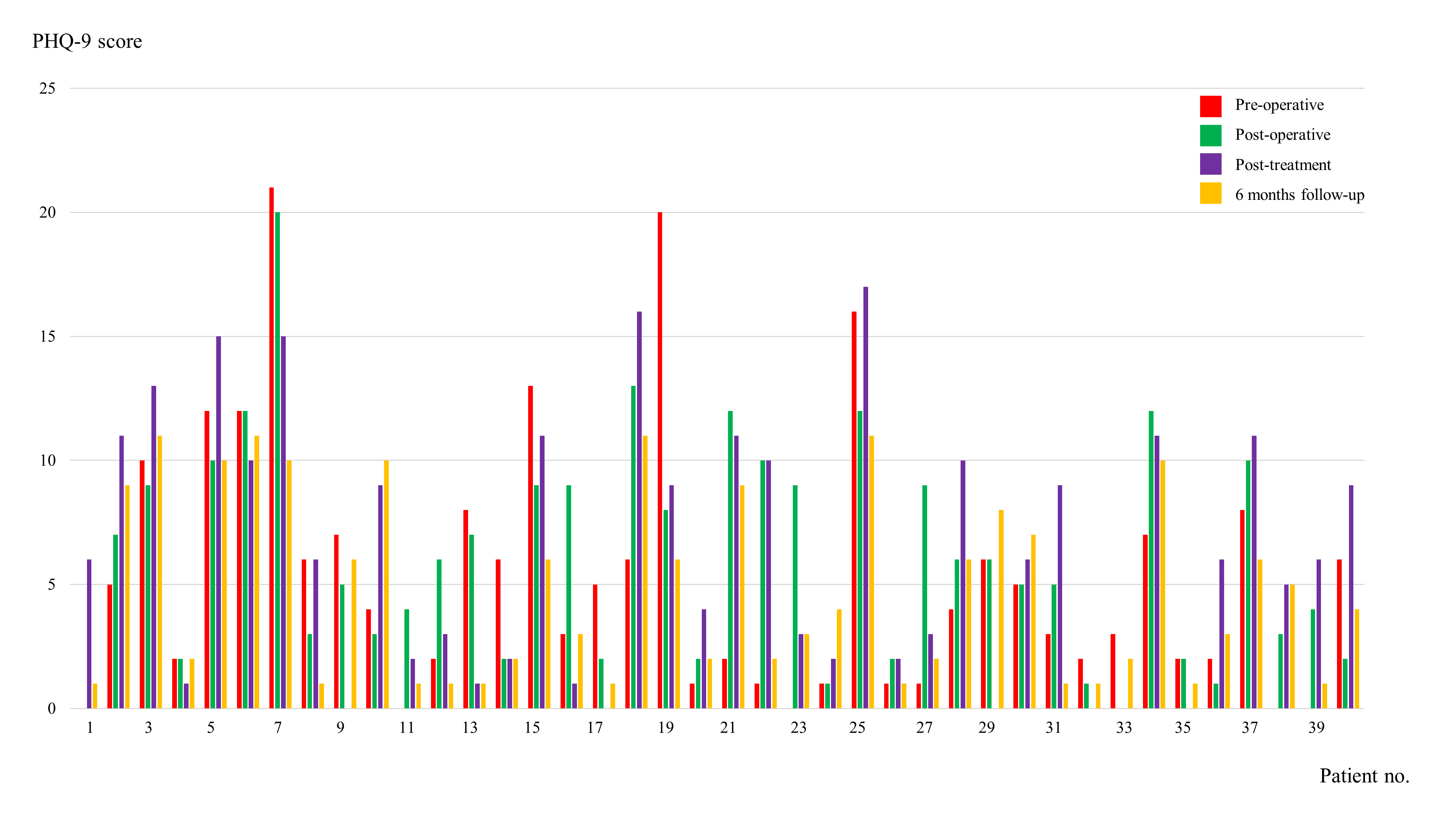

Supplement: Supplementary file 4 — Additional file 4. [file 12885_2021_8771_MOESM4_ESM.zip › Supple Figure4A (1).tif]

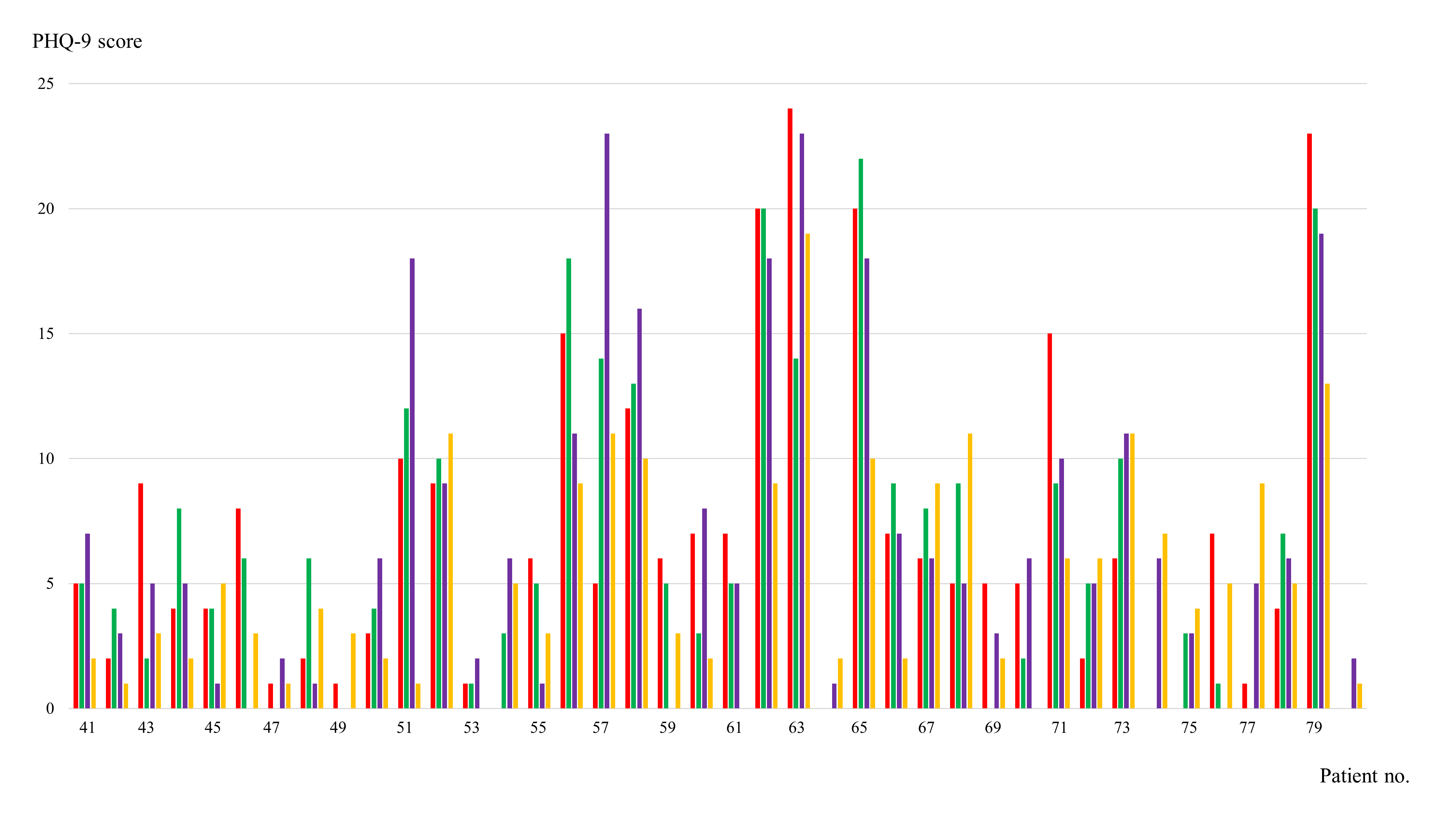

Supplement: Supplementary file 4 — Additional file 4. [file 12885_2021_8771_MOESM4_ESM.zip › Supple Figure4A (2).tif]

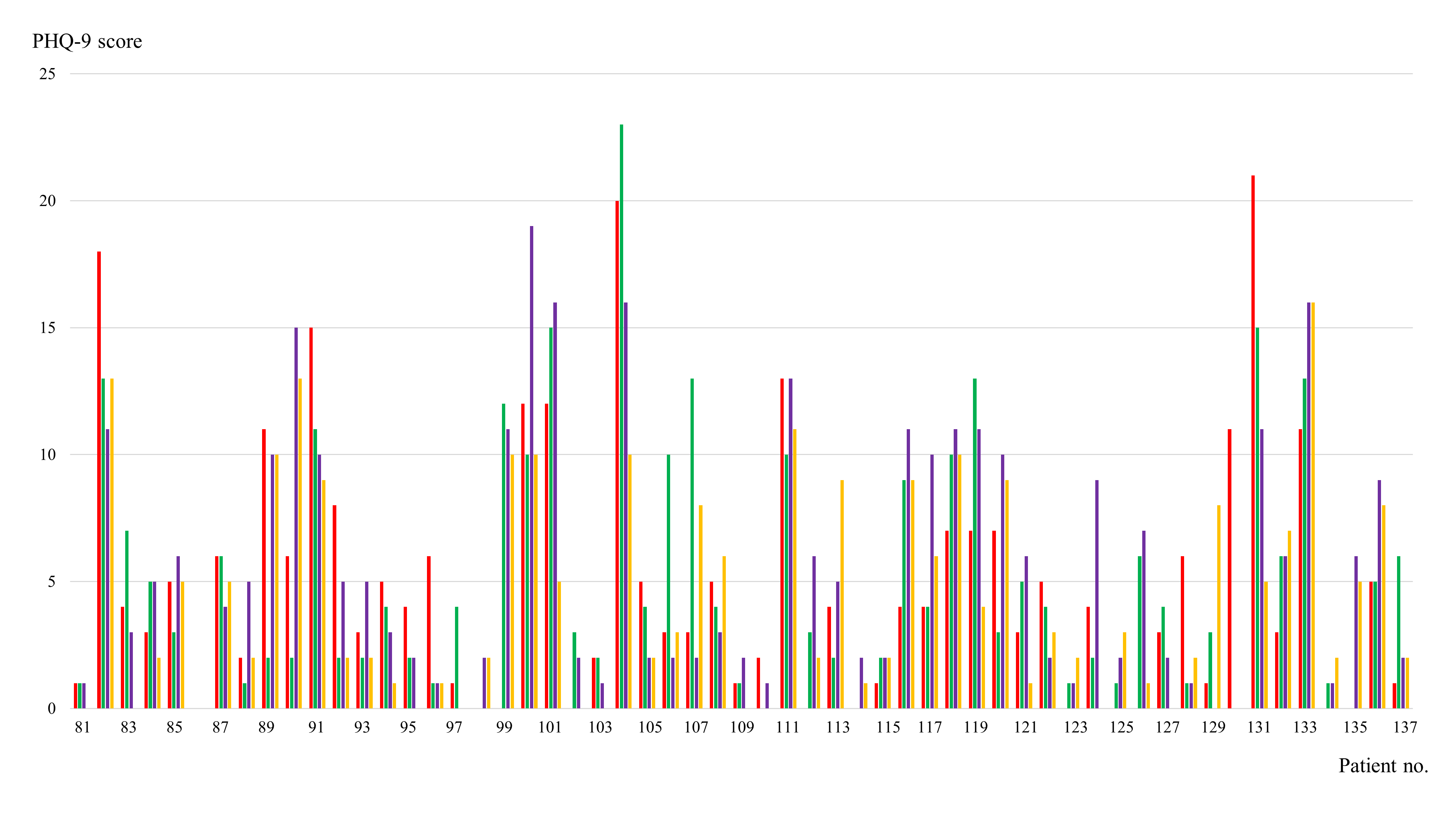

Supplement: Supplementary file 4 — Additional file 4. [file 12885_2021_8771_MOESM4_ESM.zip › Supple Figure4A (3).tif]

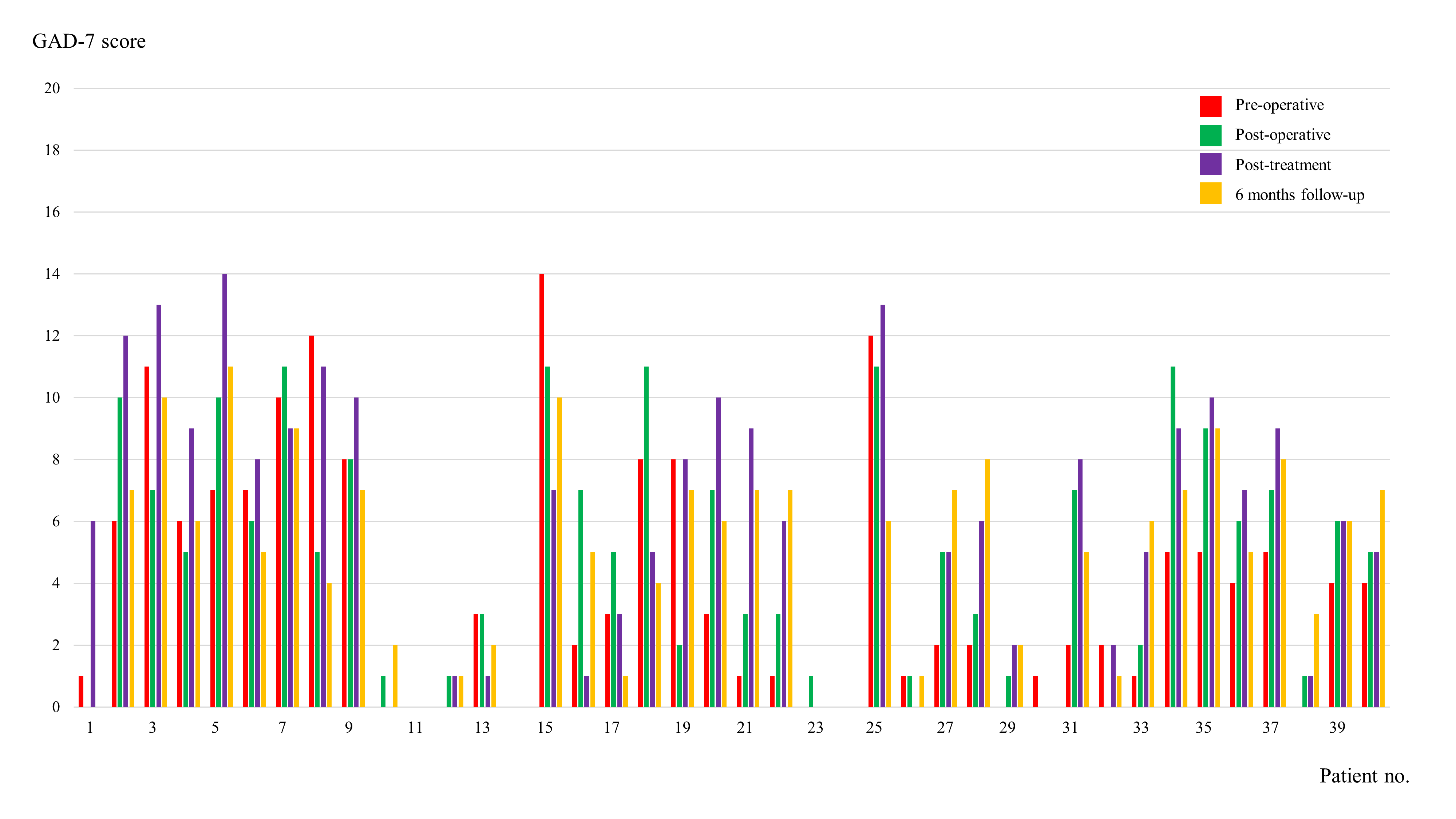

Supplement: Supplementary file 4 — Additional file 4. [file 12885_2021_8771_MOESM4_ESM.zip › Supple Figure4B (1).tif]

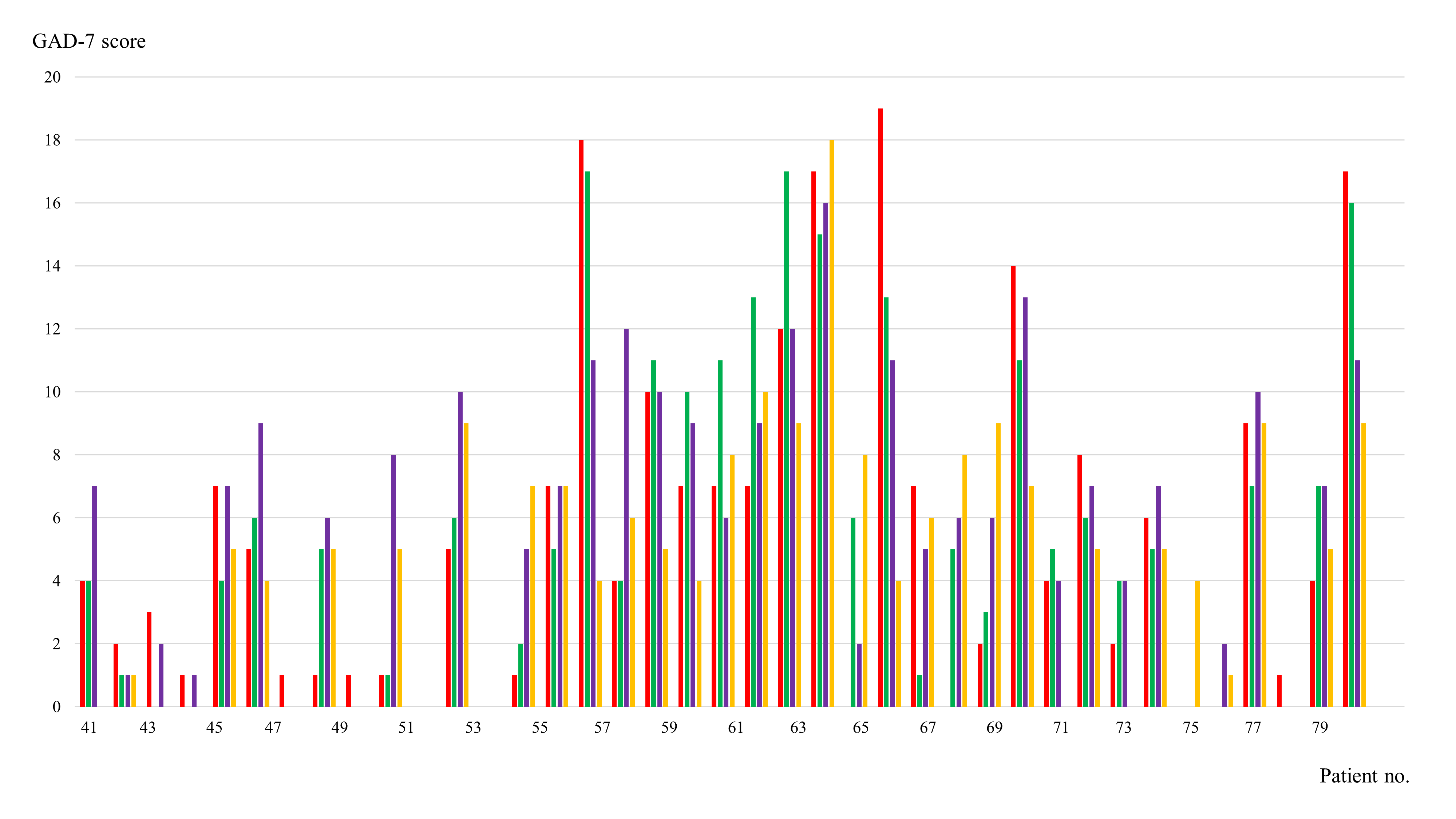

Supplement: Supplementary file 4 — Additional file 4. [file 12885_2021_8771_MOESM4_ESM.zip › Supple Figure4B (2).tif]

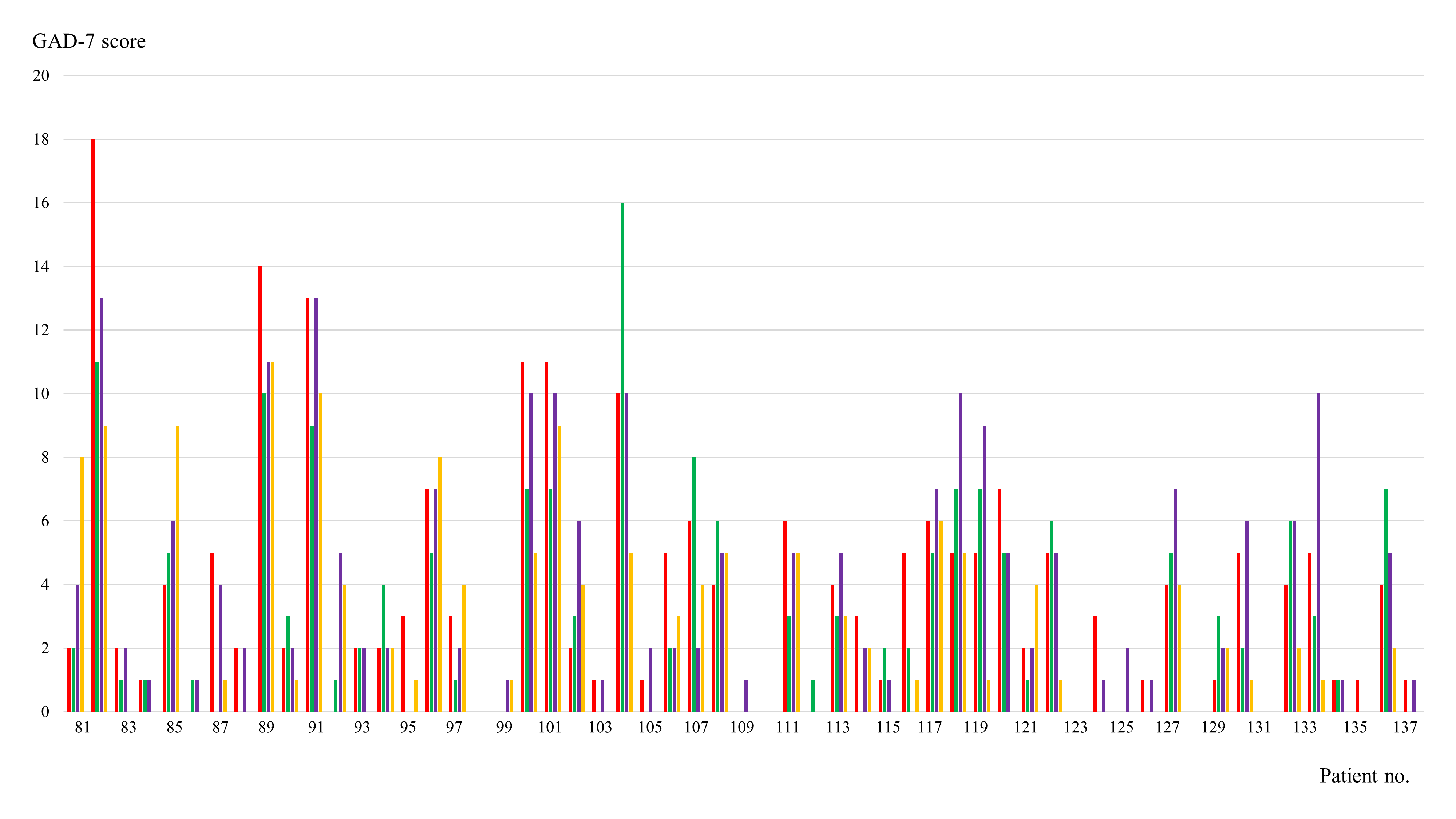

Supplement: Supplementary file 4 — Additional file 4. [file 12885_2021_8771_MOESM4_ESM.zip › Supple Figure4B (3).tif]
